# Cost‐effectiveness of combination therapy (Mechanical Diagnosis and Treatment and Transforaminal Epidural Steroid Injections) among patients with an indication for a Lumbar Herniated Disc surgery: Protocol of a randomized controlled trial

**DOI:** 10.1002/pri.1796

**Published:** 2019-07-09

**Authors:** Elizabeth N. Mutubuki, Hans van Helvoirt, Johanna M. van Dongen, Carmen L.A. Vleggeert‐Lankamp, Frank J.P.M. Huygen, Maurits W. van Tulder, Hanneke A.H.J. Klopper‐Kes, Raymond W.J.G. Ostelo

**Affiliations:** ^1^ Department of Health Sciences, Faculty of Science, Amsterdam Movement Sciences Vrije Universiteit Amsterdam Amsterdam The Netherlands; ^2^ Stichting Rugpoli Delden The Netherlands; ^3^ Department of Neurosurgery Leiden University Medical Centre Leiden The Netherlands; ^4^ Department of Anesthesiology, Centre of Pain Medicine Erasmus MC Rotterdam The Netherlands; ^5^ Department of Epidemiology and Biostatistics, Amsterdam Movement Sciences VU University Medical Center Amsterdam The Netherlands; ^6^ Department of Physiotherapy & Occupational Therapy Aarhus University Hospital Aarhus Denmark

**Keywords:** epidural injections, physiotherapy, radiculopathy

## Abstract

**Objectives:**

The general consensus is that surgical treatment is advised when conservative methods fail in patients with lumbosacral radicular syndrome (LRS). Preliminary evidence from our pilot study indicates that combination therapy (mechanical diagnosis therapy and transforaminal epidural injections) can prevent surgical treatment in patients on the waiting list for surgery. The pilot study lacked a control group, and therefore, firm conclusions pertaining to effects could not be made. This study aims to determine if combination therapy, performed while being on the waiting list for lumbar herniated disc surgery, is effective and cost‐effective compared with usual care (i.e., no intervention while being on the waiting list) among patients with a magnetic resonance imaging (MRI)‐confirmed indication for a lumbar herniated disc surgery.

**Methods:**

A randomized controlled trial will be conducted with an economic evaluation. Patients aged 18 and above with incapacitating LRS, with leg pain and an MRI confirmed indication for lumbar disc hernia surgery, will be recruited from seven Dutch hospitals. While being on the waiting list for lumbar herniated disc surgery, patients will be randomized to either the combination therapy or usual care group. The primary outcome measure is the number of patients undergoing lumbar disc surgery during 12‐month follow‐up. Secondary outcomes include back and leg pain intensity (numeric pain rating scale), physical functioning (Roland Morris Disability Questionnaires‐23), self‐perceived recovery (global perceived effect), and health‐related quality of life (EuroQol Five Dimensions Health Questionnaire (EQ‐5D‐5L) and 12‐Item Short Form Health Survey (SF‐12)). For the economic evaluation, societal and health care costs will be measured. Measurements moments are baseline, 1, 2, 4, 6, 9, and 12 months. Data will be analysed according to the intention‐to‐treat principle.

**Conclusion:**

No randomized controlled trials have evaluated the effectiveness and cost‐effectiveness of combination therapy compared with usual care in patients with an indication for lumbar herniated disc surgery, which emphasizes the importance of this study.

## 1 INTRODUCTION

Lumbar disc herniation is the most common cause of lumbosacral radicular syndrome (LRS), also known as sciatica. Characteristics of LRS include radiating lower limb pain into a particular dermatome, which may be accompanied by sensory and or motor deficits (Oosterhuis et al., 2014). Evidence suggests that the pathophysiology of LRS is not attributed to just pressure on the nerve roots but to a complex interplay of inflammatory, immunological, and pressure related processes (Stafford, Peng, & Hill, 2007). Estimated LRS incidence rate in Western countries is 5 per 1,000 (Cherkin, Deyo, Loeser, Bush, & Waddell, 1994). In the Netherlands, the incidence rate of LRS in general practice is 12 per 1,000 patients per year (Schaafstra et al., 2015). The yearly direct and indirect costs of LRS are high and estimated to be €1.2 billion in the Netherlands (Health Council of the Netherlands, 1999).

There is a lot of variation in LRS prevalence in literature (Konstantinou & Dunn, 2008). Consequently, there are disparities in spinal surgery rates regionally and internationally (Weinstein, Lurie, Olson, Bronner, & Fisher, 2006). In the United States, spinal surgery rates are 30% higher than in the Netherlands, 80% higher than in United Kingdom, and 50–60% higher than in Canada (Cherkin et al., 1994).

The Dutch guideline “Lumbosacral Radicular Syndrome” recommends surgical treatment if the radiating leg pain persists following conservative management (Kwaliteitsinstituut voor de Gezondheidszorg CBO, 2008). A significant number of patients undergoing surgery for lumbar disc herniation suffer residual complaints. Recovery rates in the literature vary wildly. Recent figures from the Netherlands suggest a rate between 69% and 79% after 2‐year follow‐up, and 10%–15% of the patients need repeated surgery, the majority of which were due to recurrent disc herniation at the same level (Arts et al., 2011). Findings of Peul et al. (2007) indicate that surgical and nonsurgical management of lumbar hernia are equally successful in the long term.

Both mechanical diagnosis and treatment (MDT) and transforaminal epidural steroid injections (TESIs) are reported to be individually effective in reducing pain and improving function among LRS patients (Chou et al., 2015; van Helvoirt et al., 2014). Epidural corticosteroid injections for radiculopathy are associated with immediate reduction in pain (Chou et al., 2015). TESIs are indicated in LRS, and the role of physiotherapy, possibly in combination with TESIs, should be further explored (Kwaliteitsinstituut voor de Gezondheidszorg CBO, 2008). None of the randomized controlled trials included by Chou et al. (2015) combined TESIs with MDT. The only two publications that we identified assessing a combination therapy of TESIs and MDT were our own pilot study (van Helvoirt et al., 2014), and a report on three cases of acute cervical radiculopathy (Desai, Padmanabhan, Simbasivan, Kamanga‐Sollo, & Dharmappa, 2012). Our pilot study suggests that combining these interventions has the potential to reduce the number of lumbar herniated disc surgeries, as only 22% of patients with a herniated lumbar disc still needed surgery after 1‐year follow‐up (van Helvoirt et al., 2014).

Research indicates that the effects of lumbar disc surgery are comparable with those of conservative treatment after 1 and 2 years (Peul et al., 2007; Peul et al., 2008). Clinical guidelines prescribe shared decision making and that pros and cons of both options should be discussed with patients (Kwaliteitsinstituut voor de Gezondheidszorg CBO, 2008; National Institute for Health and Clinical Excellence, 2016). Surgery is costly and potentially causes various side effects (e.g., nerve root damage, infection, and pain that continues after surgery); hence, spinal surgeons typically aim to prolong conservative therapy. Physiotherapists could play an important role in preventing surgery if they combine their treatment with optimal pain management (Schaafstra et al., 2015). Although the results from our pilot study seem to be promising, the effectiveness and cost‐effectiveness of a combination therapy of TESIs and MDT have not been rigorously evaluated. Therefore, this study aims to determine if a combination therapy, while being on the waiting list for a lumbar herniated disc surgery, is effective and cost‐effective compared with usual care (i.e., no intervention while being on the waiting list) among patients with a magnetic resonance imaging‐confirmed indication for a lumbar herniated disc surgery.

## 2 METHODS

### 2.1 Study design

A multicentre randomized controlled trial with a 12‐month follow‐up and a full economic evaluation.

#### 2.1.1 Ethical approval

In September 2017, the Medical Ethics Committee of the VU University Medical Centre Amsterdam approved the study protocol, registration Number NL60558.029.17 and the study was registered in the Dutch Trial Register NTR6715.

### 2.2 Subjects

#### 2.2.1 Inclusion criteria

Inclusion criteria are as follows: patients aged 18 and above; eligibility for lumbar disc hernia surgery; incapacitating LRS with leg pain (numeric pain rating scale [NPRS] > 6; with or without back pain) that had lasted for a minimum of 6 weeks with or without mild neurological deficit (i.e., Medical Research Council > 3); and a magnetic resonance imaging that confirms a hernia nuclei pulposi that compromises the spinal nerve and can explain the clinical symptoms of the patient.

#### 2.2.2 Exclusion criteria

Exclusion criteria are as follows: spine surgery and or transforaminal injections at the same level during the previous 6 months; bony stenosis; cauda equina syndrome; spondylolisthesis; pregnancy; complicated disc herniation requiring more than one operation; severe coexisting disease (e.g., osteoporosis and dementia); patient with contraindications for steroid injections; insufficient knowledge of the Dutch language; emergency surgery as determined by the neurosurgeon; and being allergic for Iohexol 240 mg/ml (i.e., OMNIPAQUE 240).

#### 2.2.3 Patient recruitment

**Figure 1:**
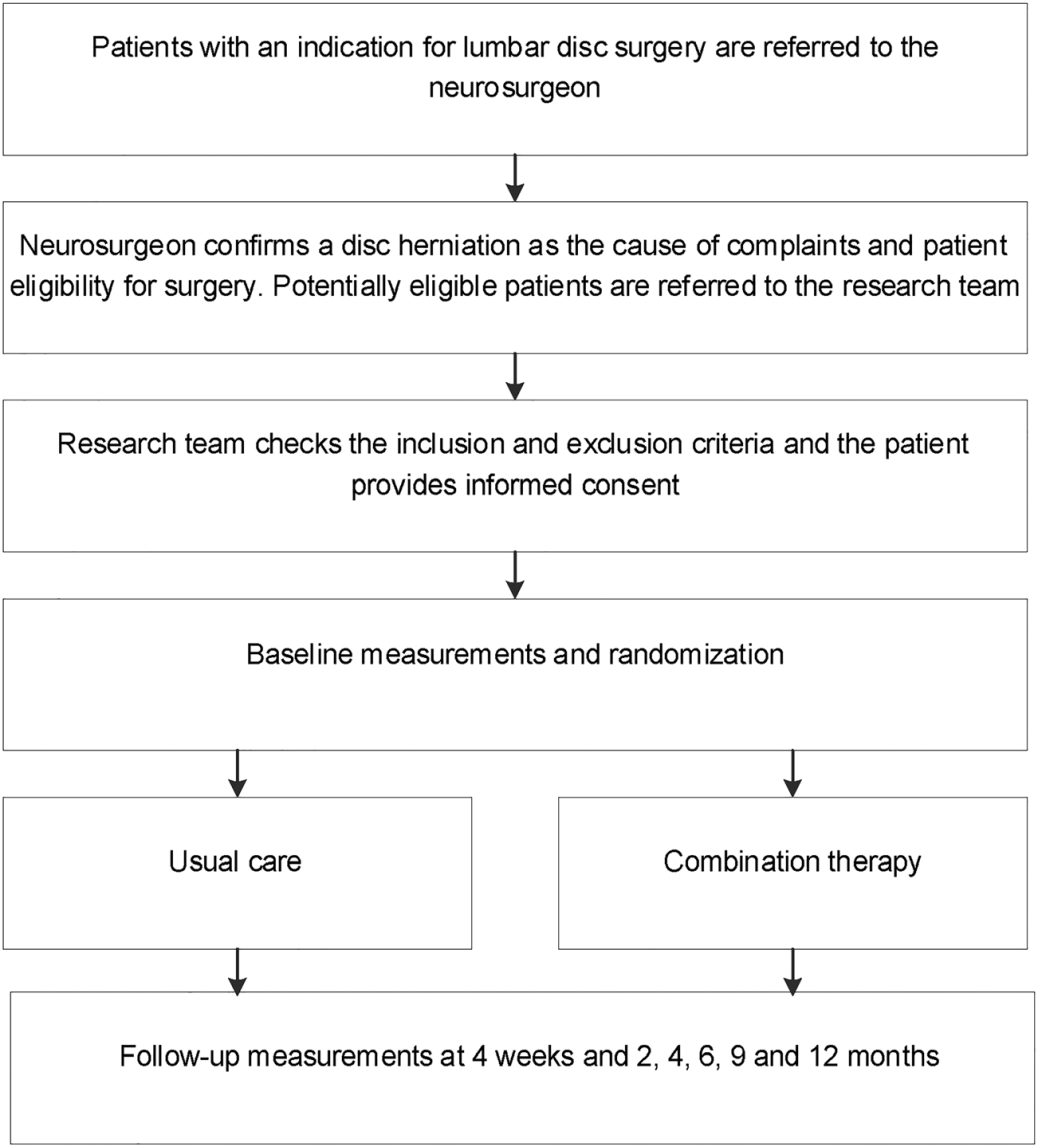
Flow chart of the Preventing lumbar disc surgery (PLUS) study

Figure 1 shows the flow diagram of the study. Neurosurgeons or orthopaedic surgeons of the participating hospitals recruit and inform the patients about the study and the possibility to participate while they are on the waiting list for surgery. The surgeon then refers the patient to the research team who will check if the patient meets the aforementioned inclusion and exclusion criteria. If the patient is eligible and gives informed consent, the patient will be included in this study. Baseline measures will then be made and patients will be randomized to either the intervention or the control arm of the study.

#### 2.2.4 Setting

Participants will be recruited from seven hospitals in the Netherlands. The hospitals were chosen due to their proximity to the four primary care‐based outpatient clinics (so‐called “Rugpoli's”) where the combination therapy will be provided.

### 2.3 Materials

#### 2.3.1 Prognostic factors

Prognostic factors measured at baseline using an online questionnaire include duration and severity of complaints before operation, various psychosocial variables (somatization, distress, and anxiety), and known confounding factors such as age, gender, educational level, and treatment expectation (Kwaliteitsinstituut voor de Gezondheidszorg CBO, 2008).

#### 2.3.2 Primary outcomes measure

The primary outcome measure is the proportion of LRS patients undergoing lumbar disc surgery during 12‐month follow‐up. Patients will be scored as either having had a lumbar surgery or not. For this purpose, patients will be asked whether they had a lumbar surgery during the previous weeks following the last assessment, using an online questionnaire at 2, 4, 6, 9, and 12 months after randomization, and validated using hospital records.

#### 2.3.3 Secondary outcomes measures

In line with the core outcome set for clinical research and clinical practice (Chiarotto et al., 2018), secondary study parameters include back and leg pain, physical functioning, and health‐related quality of life. Additionally, we will measure self‐perceived recovery, patient satisfaction (single question), and pain location (pain mannequin). Measurements will take place at baseline, 2, 4, 6, 9, and 12 months after randomization and administered through online questionnaires. An additional pain intensity measurement will be carried out at 4 weeks after randomization. The rationale is that, especially leg pain, will improve in the combination group. Complications will be noted in a case report form. Societal and health care costs will be estimated for economic evaluation using resource use data, collected through online questionnaires at baseline, 2, 4, 6, 9, and 12 months after randomization.

##### Back and leg pain will be measured using the NPRS

The NPRS ranges from 0 (“no pain”) to 10 (“representing worst pain imaginable”). The numerical pain rating scale is reliable, valid, and has good sensitivity (Williamson & Hoggart, 2005).

##### Functional status will be measured with Roland Morris Disability Questionnaires

Five Roland Morris Disability Questionnaires (RMDQ‐24) items were removed and four new items were added from the initial source of RMDQ items, to create RMDQ‐23 (Bergner, Bobbitt, Carter, & Gilson, 1981). RMDQ‐23 consists of 23 “yes” or “no” questions, measuring limitation in activity associated with back and leg pain (Kent, Grotle, Dunn, Albert, & Lauridsen, 2015). The scores can range from 0 (*no disability*) to 23 (*maximal disability*). It has been extensively used in radiculopathy and stenosis research as a standardized measure and is widely used to assess disability specific to back and leg pain making it suitable people with LRS (Kent et al., 2015). The RMDQ‐23 is regarded as reliable and valid (Yamato, Maher, Saragiotto, Catley, & McAuley, 2017). It has been translated into Dutch.

##### Health‐related quality of life (EQ‐5D‐5L and SF‐12)

The EQ‐5D‐5L is a quality of life scale that is responsive for chronic low back pain patients (Soer, Reneman, Speijer, Coppes, & Vroomen, 2012). The EQ‐5D‐5L has five health dimensions: mobility, self‐care, daily activities, pain/discomfort, and anxiety/depression. Within each dimension, the patients can self‐rate their level of severity; no, slight, moderate, severe problems, unable to perform, or do the task (Versteegh et al., 2016). For the economic evaluation, the patients' EQ‐5D‐5L health states will be converted into utility scores ranging from 0 (“death”) to 1 (“optimal health”) using the Dutch tariff (Versteegh et al., 2016).

The SF‐12 is a shorter version of the SF‐36 health‐related quality‐of‐life questionnaire. The SF‐12 has been proven to be a reliable and valid questionnaire for low back pain (Xuemei et al., 2003). The questionnaire relates to the analysis of the general functional status of patients. It consists of 12 questions from the following eight domains: (a) physical functioning, (b) physical role limitations, (c) emotional role imitations, (d) social functioning, (e) physical pain, (f) general mental health, (g) vitality, and (h) general health perception. These eight domains can be summarized into a physical and psychological main domain (Xuemei et al., 2003). For the economic evaluation, quality‐adjusted life years will be calculated by multiplying the patients' time spent in a certain health state by the respective utility value (i.e., area under the curve method).

##### Self‐perceived recovery will be measured using the global perceived effect

The global perceived effect measures a patient's self‐perceived recovery using a 7‐point scale ranging from “worse than ever” (1) to “completely recovered” (7). Being recovered will be defined as being “completely recovered” or “much improved”; other responses will be defined as not recovered. The test reliability of the global perceived effect scale is said to be good (Kampera et al., 2010).

##### Societal and health care costs

Societal costs include costs of the intervention, other health care use, informal care, unpaid productivity losses, and costs due to absenteeism (i.e., sickness absence) and presenteeism (i.e., being less productive while being at work). Health care costs will only include costs accruing to the formal Dutch health care sector.

Intervention costs will be microcosted. For this purpose, information about the combination therapy will be gathered using a case report form, including information on patient classification, number of sessions, discharge, and referral to a network MDT or to the surgeon. All other cost categories will be measured using cost questionnaires administered at baseline 2, 4, 6, 9, and 12 months after randomization. Resource use will be valued in accordance with the Dutch manual of costing (Hakkaart‐van Roijen, Tan, & Bouwmans, 2010). See Table 1: overview of the data collection.

**Table 1:** Overview of the data collection

Outcome measures	Baseline	4 weeks	2 months	4 months	6 months	9 months	12 months
Prognostic factors							
Patient demographics and prognostic factors	X						
Primary outcome							
Surgery rate			X	X	X	X	X
Secondary outcomes							
Pain leg (NPRS)	X	X	X	X	X	X	X
Pain back (NPRS)	X	X	X	X	X	X	X
Health‐related quality of life (EQ‐5D‐5L and SF‐12)	X		X	X	X	X	X
RMDQ‐23	X		X	X	X	X	X
Self‐perceived recovery (GPE)			X	X	X	X	X
Societal and health care costs (cost questionnaires)	X		X	X	X	X	X
Patient satisfaction, complications, and pain location	X		X	X	X	X	X

Abbreviation: NPRS, numeric pain rating scale; RMDQ, Roland Morris Disability Questionnaires.

### 2.4 Procedure

#### 2.4.1 Treatment allocation

Randomization will be done by an independent researcher who is not involved in treatment procedures, using a web‐based randomization program. The randomization sequence is developed centrally. Therefore, the independent researcher does not have any influence on the randomization procedure, and the treatment allocation is concealed. Patients will be randomized at the individual level and in a 1:1 ratio. We will stratify on duration of complaints (i.e., <6months vs. ≥6months), and we will use one randomization list per hospital. The randomization key will be safeguarded by an independent researcher. Patients allocated to the intervention group will be called for an appointment within 48 hr of randomization and will attend their first appointment within the first week following randomization.

#### 2.4.2 Combination therapy intervention

While being on the waiting list to receive lumbar disc surgery, intervention group participants will receive the combination therapy. The combination therapy has two parts (a) MDT and (b) TESIs and is delivered by teams of pain interventionists and physiotherapists. The pain interventionist is responsible for the TESIs and the physiotherapists for the MDT. Prior to receiving the combination therapy, patients are seen by a pain interventionist who checks for contraindications for injections and medications including steroid use. During the same appointment, participants are classified as “centralizers” or “noncentralizers” using MDT principles, that is, assessment of the patients' pain pattern responses on repeated movement tests.

##### Centralizers

Centralizers are defined as patients with centralization (i.e., a clear change in leg pain location from a more peripheral location towards a more central location, which lasts after testing staying in neutral) or directional preference (i.e., a reduction in pain intensity, but not in location, which lasts after testing staying in neutral). Testing for centralization is done according to MDT principles as described in the textbook of McKenzie and May (2003). Searching for centralization is done during repeated movement testing or sustained positioning in a certain direction. This direction differs in patients. Centralization could be found in extension, flexion, side gliding, rotation, or a combination. While testing, an MDT trained physiotherapist is able to decide how many repetitions are needed (usually between 10 and 20) and can add manual force (therapist overpressure or mobilization) if needed, depending on pain response, during and after testing. The MDT system appears to have acceptable interrater reliability for classifying patients with back pain into main/subsyndromes, when applied by therapists who have completed the credentialing examination, but unacceptable reliability in other therapists (Garcia et al., 2018). Sustained positioning is often used with high levels of leg pain and major movement loss in range of motion testing. If a certain direction is found to centralize the leg and back pain, that same direction of exercise or positioning will be used as the initial treatment direction. Patients are advised to exercise seven to eight times a day (i.e., 10 repetitions of extension in lying eight times per day). In the process of centralization, patients get postural advice in the direction of centralization (i.e., if extension is the centralizing direction, patients will be advised to keep their back in lordosis and avoid flexion movements for a period of time). As soon as centralization is full and stable, the physiotherapist trained in MDT will restore full function and most importantly check for fear avoidance of the restricted movement direction, as this could be the case after avoiding a certain direction for a period of time.

##### Noncentralizers

Noncentralizers are defined as patients with peripheralization (i.e., a clear change towards a more peripheral leg pain location or an increase in leg pain) or no effect (i.e., no change in leg pain location or intensity). The hypothesis is that in these patients, the pain is a result of the inflammation. Noncentralizers will receive a transforaminal epidural injection in accordance with the procedure described below.

##### Procedure for the TESIs

The patient lies in prone position. Under fluoroscopic guidance with contrast medium (Iohexol 240 mg/ml 0.5 cc), a very thin needle will be placed next to the compressed nerve. The contrast medium is then used to control if fluid will come to the compressed nerve. After which a combination of a local anaesthetic (lidocaine 20 mg/ml 0.5 cc) with an anti‐inflammatory agent (dexamethasone 20 mg/ml 0.5 cc) is injected. Half an hour after the injection, the pain interventionist checks the effects of the injection. The duration of pain absence is dependent on the working of the anti‐inflammatory drug on the inflammation. Two weeks after the injection, the patient is seen back by the MDT therapist to check for classification in the described subgroups and to decide if a second injection is necessary in shared decision making with patient and the pain interventionist. If pain reduction is less than 80%, then usually, a second injection will be administered with patient consent. A maximum of three injections are given to optimize pain relief.

##### Following TESIs

After the injections, participants will be reclassified using the same MDT principles, into four subgroups. The subgroups are as follows:

1. resolved symptoms (i.e., no or irrelevant pain; ≤1 on a 0–10 NPRS);
2. centralizing and significantly less pain (i.e., a pain reduction of ≥2 on a 0–10 NPRS);
3. noncentralizing and significantly less pain (i.e., a pain reduction of ≥2 on a 0–10 NPRS); and
4. noncentralizing with high levels of pain and disability (i.e., a pain score of ≥8 on a 0–10 NPRS and a disability score of >10 on the RDMQ‐23).

In the first three subgroups mentioned here, patients will get specific MDT exercises and advice. These three subgroups will be treated by an MDT therapist in one to six sessions in on average 4 weeks. If more sessions are required, patients are referred to accredited MDT therapists within the Rugpoli network. Network therapists are in close contact with the Rugpoli centres and are located all over the Netherlands.

Only the patients in Subgroup 4 will be referred back to the neurosurgeon who will assess whether patients still require surgery. Throughout the combination therapy, there is shared decision making.

#### 2.4.3 Control intervention

Control group participants will solely be placed on a waiting list and scheduled to receive lumbar disc surgery if still required. The aim of surgery is to remove the symptomatic disc herniation by a minimal unilateral transflaval approach with magnification, with the patient under general or spinal anaesthesia.

#### 2.4.4 Use of cointervention

Use of cointerventions by patients is allowed and will be monitored. Patients will be requested to complete questionnaires in which medication usage and any health care utilization is recorded throughout the follow‐up period.

#### 2.4.5 Blinding

We will not attempt to blind the patients to the intervention or control condition, as this is practically impossible in this study due to the nature of the intervention. The outcomes assessor will not be blinded, because all outcomes are self‐reported. Treatment providers (i.e., surgeons, physiotherapist, and anaesthesiologists) will not be blinded due the nature of the intervention they will provide to patients.

#### 2.4.6 Sample size

We expect that 90–95% (93% was used in the sample size calculation) of the patients in the usual care group will receive surgery, and we hypothesize that in the combination group, this rate will be reduced by 30% (or more). To detect this difference of 30% with an alpha of 0.05 (two sided), a power of 95%, anticipating a 20% drop‐out rate and taking into account the multilevel structure (with an Intraclass Correlation Coefficient (ICC) of 0.15), we need to include a total of 146 patients (*n* = 73 per treatment group; Pocock, 1983; Twisk, 2006). Even though participants will not be randomized at the hospital level, the multilevel structure of the data was accounted for in the sample size calculation, because patients are recruited from different hospitals (i.e., patients recruited from one hospital are likely to be more similar than those recruited from other hospitals) and clusters will likely not be balanced.

#### 2.4.7 Statistical/data analysis

Baseline characteristics of the patients in both study groups will be presented using descriptive statistics (mean [standard deviation], median [range], or proportion) to assess if balanced groups were obtained after randomization (i.e., having an equal distribution of the main outcome measures, prognostic factors, and known confounding factors such as age, gender, educational level, and treatment expectation).

#### 2.4.8 Primary outcome analysis

The primary analysis will be an intention‐to‐treat analysis. The primary study parameter (i.e., surgery; yes/no) will be analysed in a logistic mixed model with responses at 2, 4, 6, 9, and 12 months. In this analysis, we will take into account the levels of hospital, patient, and time of measurement. An odds ratio with 95% confidence interval between the combination therapy group and usual care group will be calculated. If necessary, the analysis will be adjusted for important prognostic characteristics.

#### 2.4.9 Secondary outcome analysis

The secondary study parameters (back pain, leg pain, self‐perceived recovery, health‐related quality of life, and functional status) will be analysed in the same way as the primary study parameter (i.e., surgery). However, for continuous outcomes, we will use a linear mixed model with the same multilevel structure.

#### 2.4.10 Economic evaluation

An economic evaluation will be performed from a societal and a health care perspective. When the societal perspective is applied, all costs and consequences relevant to the intervention will be taken into account irrespective of whom pays or benefits, whereas only those accruing to the formal Dutch health care sector will be considered when the health care perspective is applied (Hakkaart‐van Roijen, van der Linden, Bouwmans, Kanters, & Tan, 2015; Brouwer, van Exel, Baltussen, & Rutten, 2006).

The economic evaluation will be performed in accordance with the intention‐to‐treat principle and in terms of quality‐adjusted life years as well as the primary outcome proportion of LRS patients undergoing lumbar disc surgery during 12‐month follow‐up. Missing data will be imputed using multiple imputations by chained equations. The imputation model will be constructed in accordance with the recommendations of White, Royston, and Wood (2011). Imputed datasets will be analysed as specified below, after which pooled estimates will be estimated using Rubin's rules (White et al., 2011). Incremental cost‐effectiveness ratios will be calculated by dividing the difference in costs by that in effects. In order to account for the possible clustering of data, analyses will be performed using linear mixed models (Gomes et al., 2012). Accounting for the possible clustering of data (e.g., at the hospital level) is very important, as most economic evaluations fail to do so, whereas ignoring the possible clustering of data might lead to inaccurate levels of uncertainty and inaccurate point estimates (Gomes et al., 2012). Bootstrapping techniques will be used to estimate the uncertainty surrounding the cost‐effectiveness estimates. Uncertainty will be shown by plotting cost‐effect pairs on cost‐effectiveness planes and by constructing cost‐effectiveness acceptability curves (Black, 1990; Drummond, Sculpher, Torrance, O'Brien, & Stoddart, 2005; Fenwick, O'Brien, & Briggs, 2004). Cost‐effectiveness acceptability curves indicate the probability of an intervention being cost‐effective compared with a control for a range of ceiling ratios (i.e., the maximum amount of money decision makers are willing to pay per unit of effect gained).

Various one‐way sensitivity analyses will be performed to test the robustness of the study results (e.g., complete‐case analysis and per‐protocol analysis).

## 3 DISCUSSION

Prior to this study, only our pilot study has evaluated the effectiveness of a combination therapy (MDT and TESIs) in patients presenting with lumbar disc herniation (van Helvoirt et al., 2014). The pilot study indicated the importance of a multidisciplinary approach, which addresses the inflammatory and mechanical contributors to spine mediated pain, and has the potential to reduce the numbers of herniated disc surgery. However, the cost‐effectiveness of the combination therapy was not explored. The results of the pilot study should be interpreted with great caution because of lack of a control group.

The importance of this study is further emphasized by the fact that there are huge discrepancies in the treatment and management of lumbar disc herniation regionally and internationally (Weinstein et al., 2006). In addition, a lot of costs and burden to society have been associated with LRS (Health Council of the Netherlands, 1999). Therefore, the combination therapy may benefit not only individual patients but also society as a whole.

To our knowledge, this is the first randomized controlled trial investigating the effectiveness and cost‐effectiveness of the combination therapy in patients with LRS compared with usual care. Therefore, the present study aims to determine if a combination therapy, while being on the waiting list for a lumbar herniated disc surgery, is effective and cost‐effective compared with usual care among patients with an indication for a lumbar herniated disc surgery. Hence, this research will help bridge the knowledge gap in the treatment and management of patients with LRS.
